# Does Personality Have a Different Impact on Self-Rated Distraction, Job Satisfaction, and Job Performance in Different Office Types?

**DOI:** 10.1371/journal.pone.0155295

**Published:** 2016-05-25

**Authors:** Aram Seddigh, Erik Berntson, Loretta G. Platts, Hugo Westerlund

**Affiliations:** 1 Stress Research Institute, Stockholm University, SE-106 91, Stockholm, Sweden; 2 Stockholm School of Economics, Box 6501, SE-11 384, Stockholm, Sweden; 3 Department of Psychology, Stockholm University, SE-106 91, Stockholm, Sweden; University of Vienna, AUSTRIA

## Abstract

This study investigates the joint effect of office type (cell, shared room, open-plan, and flex) and personality, measured by the Big Five personality traits, on self-rated measures of distraction, job satisfaction, and job performance (measured by professional efficacy). Regression analyses with interactions between personality and office type were conducted on 1205 participants working in 5 organizations from both the private and public sectors. While few interactions were observed in the cases of professional efficacy and job satisfaction, several were observed between personality traits and office type on the level of distraction reported. Specifically, more emotionally stable participants reported lower distraction, particularly those working in flex offices. Both agreeableness and openness to experience were associated with higher levels of distraction among participants in open-plan compared to cell offices.

## Introduction

Do different office layouts suit some employees better than others? Concerns have been raised that there might be interaction effects between employees’ personalities and their surroundings, and consequently changes to office design might suit some employees more than others [1,2]. However, to our knowledge, no peer-reviewed research has tested for such interactions, although there is evidence suggesting that both personality and office type can affect employees’ productivity and appreciation of their work.

Studies have shown that certain personality traits are associated with better job performance [3,4] and job satisfaction [5]. Similarly, it has been suggested that open office layouts generate distraction and reduce job satisfaction [6], but evidence of their impact on aspects of job performance has been inconclusive [6–9]. Consequently, in this study, we examine the relationship with distraction, job satisfaction, and job performance of both office layout and personality. We examine the main effects of office type and personality, before examining interactions between office type and personality on each of the three outcomes.

### Distraction, job satisfaction, and performance in relation to personality

Models of personality [10] have been developed and used both to differentiate between people and to predict future behavioural outcomes. One of the most validated models is the Big Five model, which originally was developed through the lexical approach [11]. The Big Five is constituted of five broad traits: agreeableness, extraversion, emotional stability, openness to experience (also called imagination), and conscientiousness [12]. On each trait individuals differ along a continuum, from low to high. Highly *agreeable* people are described as trusting, altruistic, cooperative, modest, and tender-minded. *Extraverted* people, in contrast to introverted people, are described as warm, gregarious, assertive, active, excitement-seeking, and having many positive emotions. *Neurotic* people, in contrast to emotionally stable people, are anxious, angry, self-conscious, impulsive, and vulnerable. People high on *openness to experience* are described as imaginative, moved by art, emotionally sensitive, novelty-seeking, and tolerant. Finally people high on *conscientiousness* are described as competent, orderly, dutiful, motivated to achieve, self-disciplined, and as thinking before acting [13].

Associations between the Big Five personality dimensions and job performance have been the focus of several meta-analyses [cf 4]. In a review performed by Barrick and Mount [14], conscientiousness was found to consistently predict performance for all occupational groups studied, as did extraversion in jobs demanding interaction with others. Openness to experience and emotional stability were found to have some relevance for performance, although they were not as important as conscientiousness and extraversion. Agreeableness was the only trait that did not significantly predict job performance at all, although a recent study did find that individuals assessed as more agreeable reported better job performance [3].

In relation to job satisfaction, the association between Big Five personality dimensions and job satisfaction was investigated in a review article, which found that people high on neuroticism reported lower levels of job satisfaction while highly conscientious and extraverted people reported higher job satisfaction [5].

In terms of any effects of personality upon distraction, one study found that the performance of neurotic introverts, as compared to stable extraverts, was affected more by distracting stimuli [15]. More recently, evidence has been presented showing that speed of stimuli presentation impairs the efficiency of neurotics’ attention mechanisms [16]. However we were unable to find studies investigating whether stimuli in real work environments are perceived as more or less distracting depending on the personality of an employee.

### Distraction, job satisfaction, and performance in relation to office type

Associations between office type and employee distraction, job satisfaction, and performance have also been researched. A systematic literature review studying the relationship between office type and outcomes such as employee job satisfaction, privacy and performance, categorized offices according to three dimensions: location (e.g., telework office versus conventional office), layout (e.g., open layout versus cellular office), and use (e.g., fixed versus shared workplaces) [6]. Based on 49 relevant studies they concluded that there is strong evidence that open layouts reduce privacy and job satisfaction. Limited evidence suggested that open layouts intensified cognitive workload and interaction, and that room sharing improved communication [cf. 7]. Health-related outcomes may indirectly affect job satisfaction and performance, and studies have indicated benefits of cell offices in terms of lower sickness absence, fewer colds, and better self-rated health [17–20]. A recent study found that individuals working in cell offices reported higher levels of satisfaction with aspects of their environment compared with people working in shared-room or open-plan office environments [21].

In terms of distraction, a study using the same data as the present paper found that employees working in open-plan offices, compared with those working in cell and flex offices (also called hot-desking), reported more distraction [9]. In a similar vein, De Been and Beijer [7] found that employees working in cell offices and shared rooms reported better concentration, as well as more productivity support and privacy.

Seddigh et al. [9] also examined interaction effects between office type and the nature of the work tasks, finding that while most problems were reported by those who had more complex tasks in open-plan and flex offices, in cell offices the nature of work did not seem to matter for how much distraction people reported. In that study the authors also studied job performance by means of professional efficacy but no significant findings was found related to this performance outcome [9]. Seddigh et al. [22] investigated performance on complex tasks by office type, examining how memory performance was affected under normal working conditions compared with a quiet baseline (with a low amount of irrelevant stimuli) in different office types. They found a dose-response relationship between the size of open-plan office types and drop in performance, such that employees in larger offices experienced a greater drop in performance between the quiet and the normal working conditions. However, contrary to expectations, they also found that employees in cell offices had as high a drop in performance as employees in large open-plan office environments. The results from these studies suggest that the nature of work tasks together with office type can influence employees’ levels of distraction and job performance.

### Interactions of office type with personality

The studies above describe the state of evidence for the main effects of office type and personality upon a range of indicators of performance as well as distraction and job satisfaction, but interaction effects between personality and office type upon such outcomes have been little studied. One exception is research conducted by Gehlmann [23], who tested for an interaction effect of the personality trait of extraversion with three office types (cell, semi-private, and open offices) on both stress and job satisfaction. Likely due to the small sample size, 90 participants, the study failed to find any significant interaction effect between personality and office type.

Another study focused on different aspects of communication and personality traits in an open-plan office design [24]. It showed that people scoring high on extraversion, agreeableness, or conscientiousness reported being more satisfied with the communication climate and personal feedback they received in an open-plan office environments than those who scored low on each personality trait. In addition, employees with high scores for conscientiousness also reported being more satisfied with communication with supervisors in an open-plan office environment. However, this study only gathered data from employees working in open-plan office environments, which makes it difficult to draw conclusions about how these reported differences might vary by office type.

Finally Maher and von Hipple [25] found that high privacy did not protect against low job satisfaction amongst people with low stimulus screening ability [26], a trait related to emotional stability. However, the range of office types used in Maher and von Hipple [25] study was limited to two different open-plan office environments.

### The present study

Taken together, although interaction effects between office type and personality have been thought to be relevant for employee engagement and productivity, no studies with adequate designs have empirically compared employee distraction, job satisfaction, and performance in different office types in relation to personality. This study attempts to shed some light on this topic, by comparing employees working in cell, shared, open-plan, and flex offices. We aim to study whether there are any interaction effects between office types and personality traits on self-rated distraction, job satisfaction, and performance (measured by professional efficacy).

For most of the personality traits, one can hypothesise both positive and negative effects upon the outcomes depending on the interaction between office type and personality. For example, given that neurotic people may become anxious more easily [13], the unpredictable nature of the open-plan office environment might cause people high on neuroticism to report lower job satisfaction if they work in open-plan offices. However, an opposite effect is also possible, namely that neurotic people might report higher job satisfaction in open-plan office environment due to feeling more supported by having co-workers around. In a similar vein, irrelevant stimuli in the surroundings might disrupt the structured approach of conscientious people more, leading them to report higher level of distraction. However, equally, given their more structured approach, conscientious people might be more effective than less conscientious people at finding strategies to cope with the distractions generated by open-plan office environments.

Given the lack of strong theories supported by empirical data, we have chosen to pose a research question rather than formulate specific hypotheses: Do personality traits interact with office type on self-rated distraction, job satisfaction, and job performance and, if so, how?

## Material and Methods

### Study design and sample

This study employed a cross-sectional design to investigate the combined effect of office type and personality traits on three self-reported outcomes: distraction, job satisfaction, and performance (measured by professional efficacy).

Researchers invited organisations to participate and selected the offices based on a hierarchical sampling procedure which aimed to include organizations from different labour market sectors. Five organisations were selected and accepted to participate in the study. Three of the employers develop and/or support high-tech products, supplying IT solutions or providing full life-cycle IT services. The fourth employer works in construction and property development, while the last employer, a public sector organization, is the largest placement service for job-seekers in Sweden. Within these organizations, departments and office buildings with various office types were selected in an effort to include a sufficient number of employees in each studied office type within these organizations. In order to make the data collection time and cost-effective, offices with less than 50 employees were not included in the study. An exception was made to invite employees from a division within the public sector organization containing about 500 employees scattered in various locations, not all of which were visited by the researchers. Departments in the process of changing the physical design of their offices were not included in the study.

Before sending out the electronic link to the survey, the research team obtained lists of those employees whose managers had stated that they had been working fewer than 3 months at the workplace, that they spent less than 50% of their working time in the office or that they had a designated workstation but spent less than 25% of their working time at their workstation. Workstation refers to the individual work area that is designated to the specific employee. These individuals were excluded to ensure that the employees had enough experience to form an opinion about their performance in the studied office environment. The sample consisted of 2087 professionals or higher grade white-collar employees who were invited to answer a web survey, of which 1445 (69%) completed the entire survey. For the full list of the 336 items in the survey, please see Seddigh [8]. Of those invited to complete the survey, 155 respondents were subsequently removed as they stated that they had recently changed workstation, and another 157 people were removed due to taking long period of vacation or parental leave. The sample size in the main analysis was 1205 after these exclusions and additionally excluding 19 individuals with more than two items missing for any of the personality traits.

Employees in the five organizations were informed in writing and verbally during information meetings that their participation in the study was voluntary. They were asked to fill in the survey only if they agreed to participate in the study. Employees were also informed that they could leave the study by contacting the researchers even after partially or fully completing the survey. The Regional Ethical Review Board in Stockholm (Regionala etikprövningsnämnden i Stockholm), approved the study and consent procedure. The study was conducted in accordance with the American Psychology Association’s ethical standards.

### Measures

Office type. Respondents’ physical environments were categorized into the following 4 office types: cell offices containing one individual workstation, shared offices containing 2–3 individual work areas, open-plan offices containing at least 4 individual workstations, and flex offices for employees who did not have an individual workstation in the office (regardless of the spatial layout of the office) and can choose where in the office environment they want to work [9,19,22]. Cell offices had walls surrounding the individual workstation. All cell and shared room offices had a door that could be closed and were also located around the perimeter of the buildings, having good access to windows. Employees in open-plan offices had windows in sight but their individual work areas were placed at varying distances from the windows.

Before sending out the survey, the first author visited most of the office buildings and the positions of respondents’ individual work areas were plotted on architectural drawings in order to categorize respondents’ offices. Those respondents whose offices had not been mapped by the researcher were categorized from their responses to the following 3 web survey questions: ‘At your workplace, do you have your own room or do you share a room with others’, ‘At your workplace, do you have your own workstation’, ‘If you share a room with others, stand up and count how many workstations you see from your own workstation’. If the respondent responded that they did not have their own workstation, they were classified as working in a flex office.

For 996 participants, both researcher and e-survey responses were available: these corresponded in 76% of cases. The majority of the mismatched cases were due to differences between assessments in terms of the size of the open-plan office type (88%) (however, all sizes of open-plan offices have been grouped together in this study); only in 46 cases (12%), was the disagreement more substantial. Where there was lack of agreement, the first author’s classification was used [also see 9].

Personality traits. The Big Five personality traits were measured by the Swedish version of the 50-item International Personality Item Pool (IPIP) [27]. Five traits: agreeableness (Cronbach’s α = .80, sample item “Make people feel at ease”), emotional stability (Cronbach’s α = .87, sample item “Get stressed out easily”), openness to experience (Cronbach’s α = .77, sample item “Am full of ideas”), extraversion (Cronbach’s α = .87, sample item “Don’t mind being the centre of attention”), and conscientiousness (Cronbach’s α = .75, sample item “Pay attention to details”), were measured, each with 10 likert-scaled items ranging from ‘Very inaccurate’ (1) to ‘Very accurate’ (5).

*Distraction* was measured by four items using a five-point rating scale (1 = never, 5 = very often or 1 = to a small extent, 5 = to great extent) [9]. For example, one of the questions stated “How often are you for some reason disturbed so that you do not get the opportunity to fully immerse yourself in the task you have in front of you?” Cronbach’s α coefficient of internal reliability was .83, indicating satisfactory consistency.

Job satisfaction. Three items were used to measure job satisfaction, with one example item being: “I feel satisfied with my work” (ranging from 1 = do not agree, 5 = agree completely) (Cronbach’s α = .93) [28].

*Job performance* was operationalized using the *professional efficacy* subscale (6 items) of the Swedish version of the Maslach Burnout Inventory–General Survey (MBI-GS) was used as an indicator of employee performance with one example item being: “In my opinion I am good at my job” [29]. All items were scored on a 7-point rating scale (ranging from 1 = never, 7 = daily) (Cronbach’s α = .83).

Covariates. The covariates included in the models were age (continuous variable), labour market sector (0 = public, 1 = private), sex (0 = male, 1 = female), and educational level (dichotomized: 0 = low, for those without an academic degree; 1 = high, for those with an academic degree).

### Data analysis

Each outcome variable—distraction, job satisfaction, and professional efficacy—was analysed in a separate set of models. Descriptive statistics are presented in Table 1 and correlations between the variables within each office type are presented in S1–S4 Tables. Multiple regression analysis was performed in order to examine the effects of office type and personality on self-rated distraction, job satisfaction, and professional efficacy (Table 2). For the regression analyses, the scores for the five personality types were centred at their sample means in order to aid interpretation of the personality-office type interactions. In each table, Model 1 presents the results for the control variables: gender, age, education, and sector, in relation to each of the three main outcomes. In Model 2, the main effects for office type and the personality traits are displayed. Model 3 additionally presents the interactions between office type and the personality traits. R-squares, changes in R-squares, and results from F-tests of the significance of R-square changes are reported.

**Table 1:** Description of the respondents by office type (*N* = 1205).

Office type	n	Female sex %	Mean age in years (SD)	Educational level (high) %	Private sector %	Agreeable-ness (SD)	Emotional stability (SD)	Openness (SD)	Extraversion (SD)	Conscientiousness (SD)
Cell	275	71.3	48.9 (10.6)	79.6	21.8	4.2 (.4)	3.7 (.6)	3.5 (.4)	3.3 (.6)	3.8 (.4)
Shared	89	73.0	45.9 (9.9)	87.6	7.9	4.2 (.4)	3.7 (.6)	3.6 (.4)	3.3 (.6)	3.8 (.5)
Open-plan	784	57.8	47.1 (10.8)	69.5	32.3	4.1 (.5)	3.7 (.6)	3.5 (.5)	3.3 (.6)	3.8 (.5)
Flex	57	45.6	47.1 (9.9)	82.5	66.7	4.1 (.4)	3.7 (.6)	3.5 (.5)	3.3 (.5)	3.7 (.4)
Total	1205	61.4	47.4 (10.6)	73.8	29.7	4.1 (.4)	3.7 (.6)	3.5 (.5)	3.3 (.6)	3.8 (.5)

**Table 2:** Distraction, job satisfaction and professional efficacy in relation to the main effects and interactions of office type and Big Five personality traits, *N* = 1205, unstandardized beta weights.

	Distraction			Job satisfaction			Professional efficacy		
	M1	M2	M3	M1	M2	M3	M1	M2	M3
Sex									
Male	*Ref*.	*Ref*.	*Ref*.	*Ref*.	*Ref*.	*Ref*.	*Ref*.	*Ref*.	*Ref*.
Female	.07	.06	.05	.14*	.16**	.15*	.05	.02	.02
Age	–.00	.00†	.00†	.00	–.00	–.00	.00	.00	.00
Educational level									
Low	*Ref*.	*Ref*.	*Ref*.	*Ref*.	*Ref*.	*Ref*.	*Ref*.	*Ref*.	*Ref*.
High	.03	.06	.06	–.10	–.05	–.03	–.06	–.03	–.02
Sector									
Public	*Ref*.	*Ref*.	*Ref*.	*Ref*.	*Ref*.	*Ref*.	*Ref*.	*Ref*.	*Ref*.
Private	.12*	.11*	.13*	–.07	–.07	–.07	–.06	–.10†	–.10†
Cell		*Ref*.	*Ref*.		*Ref*.	*Ref*.		*Ref*.	*Ref*.
Shared		.66***	.67***		–.08	–.05		.14	.18†
Open-plan		.88***	.87***		.05	.06		.13*	.14*
Flex		.73***	.78***		.05	–.00		.10	.08
Agreeableness		.20***	–.07		.11	.07		.23***	.21
Agreeableness*Cell			*Ref*.			*Ref*.			*Ref*.
Agreeableness*Shared			–.03			.05			–.47†
Agreeableness*Open-plan			.36*			.08			.08
Agreeableness*Flex			.55†			–.50			.11
Emotional stability		–.43***	–.25**		.44***	.42***		.33***	.35***
Emotional stability*Cell			*Ref*.			*Ref*.			*Ref*.
Emotional stability*Shared			–.25			.10			–.04
Emotional stability*Open-plan			–.20†			–.00			–.04
Emotional stability*Flex			–.41*			.21			.15
Openness to experience		.15**	–.11		–.02	–.17		.16**	.19
Openness to experience*Cell			*Ref*.			*Ref*.			*Ref*.
Openness to experience*Shared			.34			–.18			.31
Openness to experience*Open-plan			.34*			.18			–.09
Openness to experience*Flex			–.16			.55†			.40
Extraversion		–.04	–.02		.02	–.05		.08*	.21*
Extraversion*Cell			*Ref*.			*Ref*.			*Ref*.
Extraversion*Shared			–.07			.20			–.12
Extraversion*Open-plan			–.02			.08			–.16
Extraversion*Flex			–.18			.10			–.19
Conscientiousness		.05	–.07		.17**	.42**		.40***	.54***
Conscientiousness*Cell			*Ref*.			*Ref*.			*Ref*.
Conscientiousness*Shared			.28			–.13			–.10
Conscientiousness*Open-plan			.13			–.32*			–.19
Conscientiousness*Flex			.59†			–.60†			–.33
Constant	3.25***	2.26***	2.28***	3.82***	3.90***	3.87***	5.74***	5.71***	5.68***
*R*^2^	.01	.24	.26	.01	.11	.12	.00	.18	.20
*∆R*^2^		.23***	.02†		.10***	.01		.18***	.01

*** *p* < .001

** *p* < .01

* *p* < .05

† p < .10.

## Results

Table 1 displays the demographic characteristics of the respondents by office type. The make-up of offices varied in terms of the covariates, particularly gender and public/private sector; the mean scores for the Big Five personality dimensions were, however, similar across the 4 office types. Correlations between the personality and outcome variables within each office type are shown in the S1–S4 Tables.

The main effects of office type and personality upon the three outcomes of distraction, job satisfaction, and professional efficacy are displayed in Table 2. Out of the Big Five personality traits, only agreeableness, emotional stability, and openness to experience were associated with distraction in the mutually adjusted model: participants who were more agreeable, less emotionally stable, and more open to experience reported more distraction (Table 2, Model 2). More emotionally stable and conscientious people reported greater job satisfaction while all personality traits were associated with perceived professional efficacy. Office type was significantly associated with the distraction outcome only, such that, compared to participants working in cell offices, those working in shared, open-plan, and flex offices reported more distraction. Office type was not associated with job satisfaction or professional efficacy.

**Fig 1:**
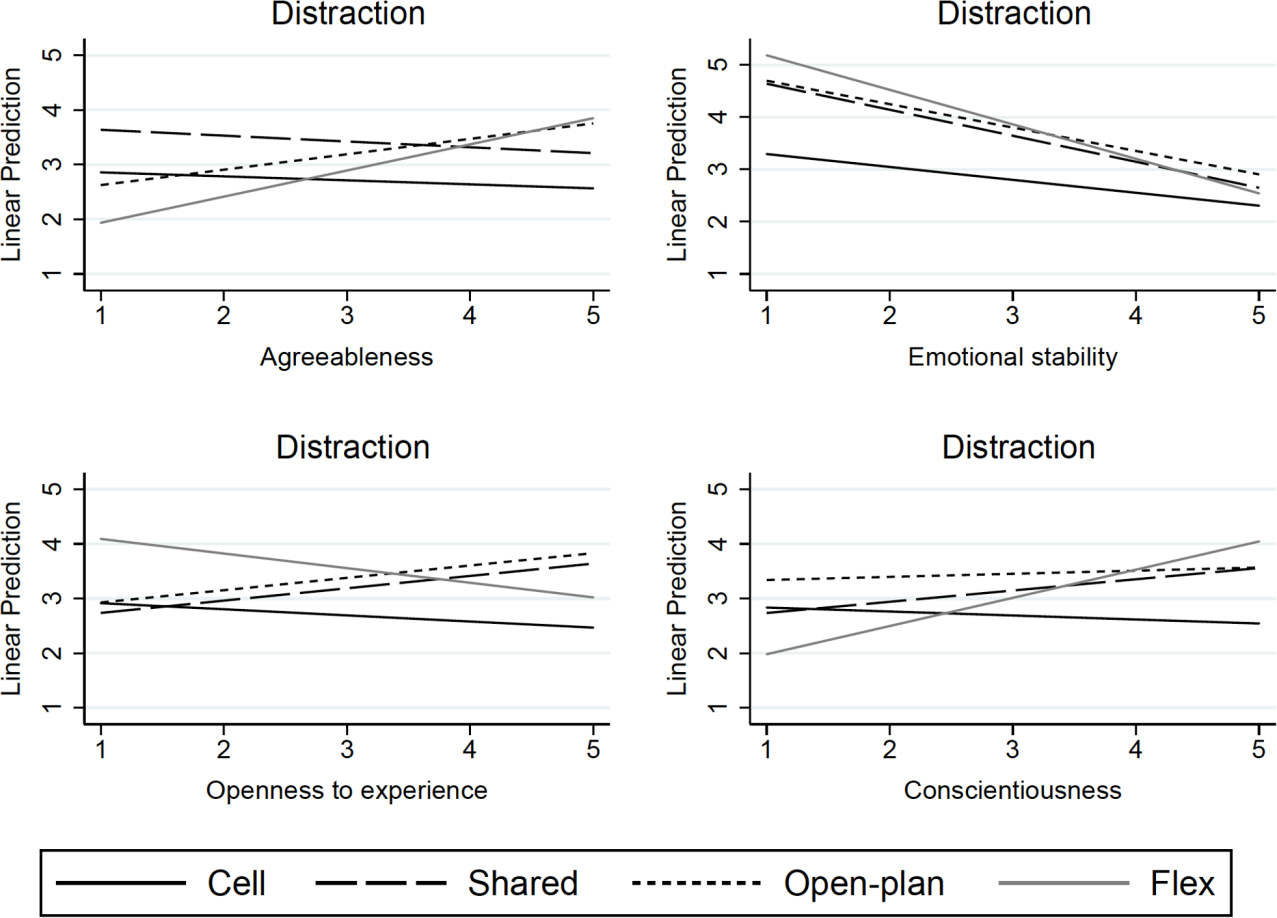
Adjusted predictions of office type upon distraction in relation to selected personality traits in 1205 Swedish public and private sector workers.

Model 3 in Table 2 displays results for the analyses of interaction effects between office type and personality for each outcome. There were several significant interactions concerning **distraction** (cf. Fig 1). While no relationship was observed between agreeableness and distraction among employees in cell offices (*b* = –.07, *p =* .589), compared to this non-significant relationship, greater distraction was reported by more agreeable participants who were working in open-plan offices (*b* = .36, *p* = .018) and the pattern was similar, but at a marginal level of significance only, for flex offices (*b* = .55, *p =* .084). More emotionally stable employees working in cell offices reported lower distraction (*b* = –.25, *p =* .007), an effect which was accentuated among those in flex offices (*b* = –.41, *p =* .040) and, at marginal significance, among those in open-plan offices (*b* = –.20, *p =* .051). While employees in cell offices did not differ significantly in how distracted they were depending on the trait openness to experience (*b* = –.11, *p =* .356), employees high on openness working in open-plan offices reported more distraction (*b* = .34, *p =* .013). Similarly, while no relationship was observed between conscientiousness and distraction among employees in cell offices (*b* = –.07, *p =* .517), more conscientious employees in flex offices tended to report higher distraction at a marginal level of significance (*b* = .59, *p =* .051). Interactions were not found between extraversion and office type in relation to distraction.

**Fig 2:**
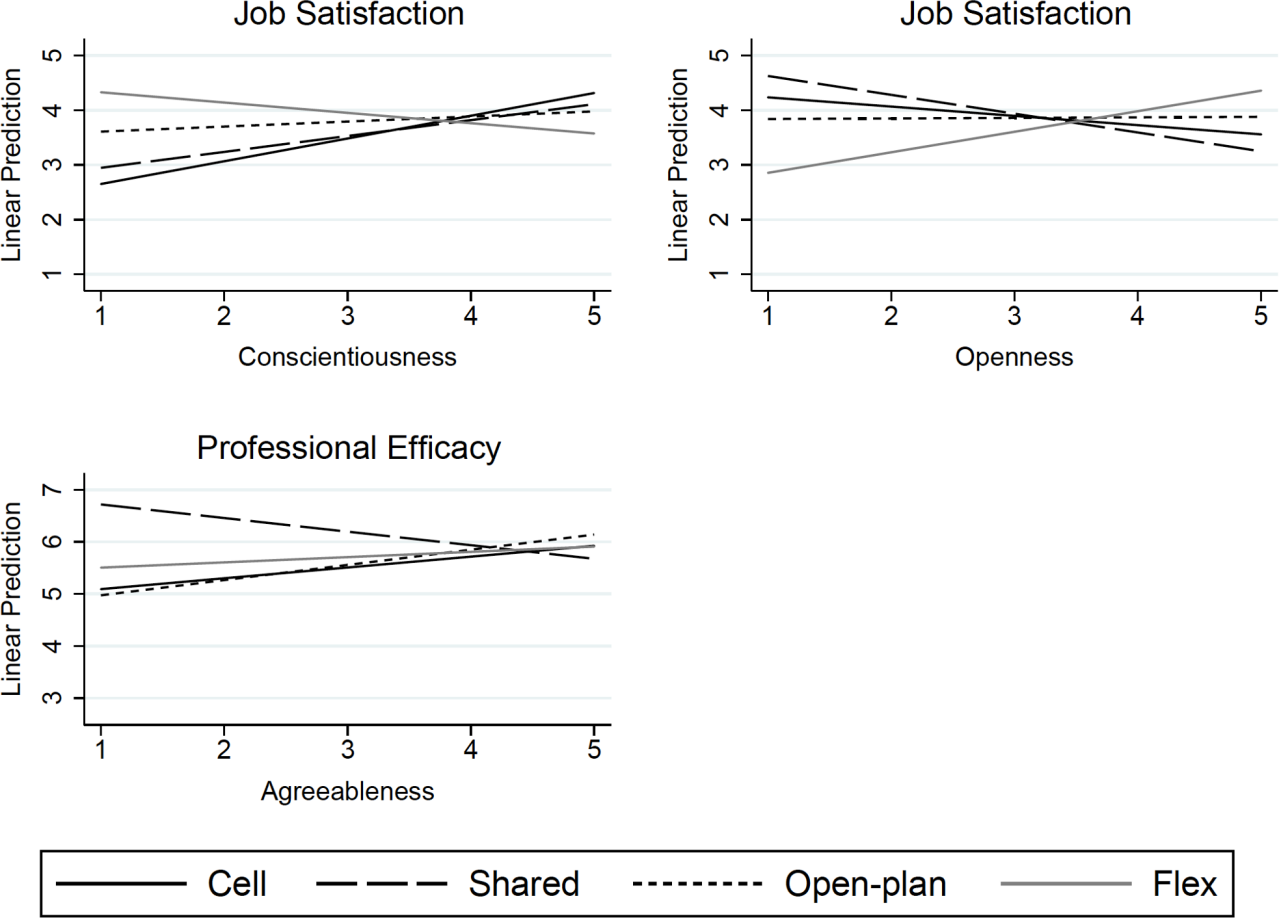
Adjusted predictions of office type upon job satisfaction and professional efficacy in relation to selected personality traits in 1205 Swedish public and private sector worker.

Concerning **job satisfaction**, in general, interaction effects were not observed. An exception was a significant interaction between conscientiousness and office type (Fig 2). While more conscientious employees in cell offices reported greater job satisfaction (*b* = .42, *p =* .001), this effect was substantially smaller among employees working in open-plan offices compared to the reference group working in cell offices (*b* = –.32, *p =* .027) and, in addition, there was a reversal among those working in flex offices at a marginal level of significance (*b* = –.60, *p =* .075). In the case of openness to experience, employees working in cell offices reported similar levels of job satisfaction regardless of their level of openness to experience (*b* = –.17, *p =* .216), while employees working in flex offices who were more open to experience tended to report greater job satisfaction at a marginal level of significance (*b* = .55, *p =* .068) (Fig 2). Emotional stability was associated with higher job satisfaction, with no difference depending on office type.

In relation to **professional efficacy**, no significant interaction effects were observed at the 95% level of significance. However, compared to the lack of relationship between agreeableness and professional efficacy observed for employees working in cell offices (*b* = .21, *p =* .116), more agreeable employees in shared offices tended to report lower levels of professional efficacy at a marginal level of significance (*b* = –.47, *p =* .085) (Fig 2). Emotional stability, extraversion, and conscientiousness were associated with higher professional efficacy with no difference between office types.

## Discussion

In this study we investigated whether the relationship of office type with the outcomes distraction, job satisfaction, and job performance (measured as professional efficacy) differed depending on the participants’ personality traits. The results of the present study provide partial support for the presence of such interaction effects, largely in the case of the distraction outcome.

Specifically, the positive associations between distraction and the personality traits of agreeableness and openness were stronger among employees working in open-plan offices than among those in cell offices. A similar trend was perceived for employees working in flex offices where, in comparison to those in cell offices, agreeable and conscientious employees tended to report more distraction. Furthermore, although high emotional stability seemed to buffer against self-reported distraction in general, this was accentuated among employees working in flex offices and possibly also those in open-plan offices.

A possible explanation for the interactions observed between agreeableness and office type in relation to distraction might be that highly agreeable people tend to be less keen to assert themselves by expressing their needs. This means that, in open-plan and flex offices where expressing the need not to be disturbed might be important in order to avoid distraction, more agreeable people may be disadvantaged because of their tendency not to prioritise their own needs. Employees high on openness are more curious and might therefore focus more on external stimuli, which could explain why they get more distracted than if they had worked in cell offices where the exposure to other people’s conversations and other environmental stimuli is lower. Emotional stability on the other hand might decrease the attention directed towards possible threats in the surroundings, which may lead to lower perceived distraction.

However, little evidence was found for interactions between personality and office type affecting job satisfaction. Job satisfaction was affected only by the interaction between conscientiousness and office type: while conscientious employees in general reported higher job satisfaction, this advantage was smaller among employees working in open-plan compared with cell offices. This could be due to the level of control individuals need over their environment. Conscientious people might perceive open-plan environments and flex offices as less predictable and more chaotic than cell offices in which employees have greater control over their surroundings. Turning to job performance (measured by professional efficacy), no interaction effects, significant at the 95% significance level, were found.

Most of the explained variance is explained by the main effects of personality and office type and not by interaction effects between office type and personality. Therefore, even if the interaction effects are significant, our findings suggest that office type and personality are largely independent predictors of the outcomes studied in this paper. Although personality in general seems to be related to employee job performance, the joint effects of personality traits and office type are only modest at best.

### Strengths, limitations, and future research

This study has several strengths. First, it is original in differentiating between and comparing different office types to each other. This enables conclusions to be drawn about the relationships between office type and the outcomes that are not confounded by personality traits.

Second, we have rich information about both the sample and the workplaces included in the study. This made it possible to exclude irrelevant cases, for example those who were recently employed or spent little time in the office, strengthening the validity of the analyses.

Third, although much of the data were obtained by a survey, in most cases it was possible to use objective classifications of workstations to categorize participants into appropriate office types, reducing although not entirely eliminating the possibility of common method bias [30]. For future studies, it would be preferable to separate measures of personality from outcomes in time.

Fourth, findings relating to the main effects between personality and measures of job performance (measured by professional efficacy) and job satisfaction were in line with results from previous studies, which supports the validity of the findings. Specifically, participants who scored highly on agreeableness, extraversion, emotional stability, openness to experience, and conscientiousness tended to report higher professional efficacy and job satisfaction, as has previously been reported [3,4,14,25].

Several limitations should be discussed. First, only a few significant interaction effects between office types and personality traits on job satisfaction and professional efficacy were found. However, interaction effects are hard to detect in field studies, even where they actually exist [31]. Therefore, the negative findings may have resulted from Type II error in which interaction effects that do exist were not observed. Although the sample is moderately large, at 1205 participants, this possibility cannot be excluded. In addition, the study sample contains participants from only five organizations, so more research on a different sample would be required to confirm these findings.

Second, the data in the present study concerning personality traits and the outcome variables were assessed by a web questionnaire. Consequently, there is a risk of common method bias [30]. However, the researchers have in most workplaces conducted the assessment of office type through objective categorization, rather than relying on participants’ own classifications. A previous study based on the same sample reported quite a good correspondence between the researchers’ and respondents’ descriptions of their office type [9], which means that the assessment of office type is likely to be unbiased.

Third, none of the companies that we included had all the office types of interest. Therefore it was not possible to confirm that similar trends as observed in the total sample were observable within each company. In addition, since the employers in the study do not constitute a random sample, it is possible that employers agreed to participate in the study only if they considered that they did not have major problems within their organization. This might have affected our results in such a way that the observed interaction effects might only be generalizable to office environments that are well functioning.

Fourth, this study applies a cross-sectional design, which makes it impossible to draw conclusions about causality. Further work gathering data from the same individuals before and after exposure to certain office environments would provide useful contributions by examining the temporal relationships between exposure and outcome.

Fifth, this study used self-ratings of professional efficacy to measure job performance. Although professional efficacy has been used as an indicator of job performance in previous studies [9], future studies should include a broader range of performance measures.

A final limitation is the possibility of selection bias. If office type influences where people choose to work, those with certain preferences may cluster in certain office types. Although personality traits were evenly distributed across office types (Table 1), unmeasured factors related to selection bias may still affect the outcomes.

## Future Research

This study investigated the interaction effect of personality and office type on distraction, job satisfaction, and job performance (measured by professional efficacy) with an explorative approach. In order to confirm our findings, future research should by a hypothesis-testing approach investigate whether these findings can be replicated.

## Conclusion

The present study looked for interactions between office type and personality on distraction, job satisfaction, and job performance. We were unable to show that individuals with different personalities responded differently to different office types in terms of self-reported job satisfaction, and professional efficacy. Where interactions were found, in relation to self-reported distraction only, their contribution to explaining variability in distraction was small. This is the first peer-reviewed study to describe such interactions with personality across different office types. We would advise interested employers that the scientific evidence for different office layouts suiting employees with certain personalities better than others is poorly established. Other factors, such as nature of work tasks, are likely to be more important.

## Supporting Information

S1 Table Correlations between the personality traits and outcome variables (*n* = 275), cell office type. (PDF)

S2 Table Correlations between the personality traits and outcome variables (*n* = 89), shared office type. (PDF)

S3 Table Correlations between the personality traits and outcome variables (*n* = 784), open office type. (PDF)

S4 Table Correlations between the personality traits and outcome variables (*n* = 57), flex office type. (PDF)
