# Supplementary material for: Does Personality Have a Different Impact on Self-Rated Distraction, Job Satisfaction, and Job Performance in Different Office Types?
Source: PLoS One. 2016 May 25;11(5):e0155295. doi: 10.1371/journal.pone.0155295 (PMC4880328; doi:10.1371/journal.pone.0155295)
Supplement: S1 Table — (PDF) [file pone.0155295.s001.pdf]

S1 Table. Correlations between the personality traits and outcome variables (*n*=275), cell office type.

	1.	2.	3.	4.	5.	6.	7.	8.	9.	10.	11.	12.
1. Agreeableness	1.00											
2. Emotional stability	.14*	1.00										
3. Openness to experience	.25***	.20**	1.00									
4. Extraversion	.24***	.30***	.29***	1.00								
5. Conscientiousness	.24***	.11†	–.03	.08	1.00							
6. Distraction	–.06	–.21***	–.10†	–.11†	–.08	1.00						
7. Job satisfaction	.08	.24***	–.05	.04	.25***	–.16**	1.00					
8. Professional efficacy	.23***	.32***	.18**	.27***	.33***	–.09	.47***	1.00				
9. Gender (female)	.18**	–.18**	–.17**	–.03	.18**	.02	.15*	.01	1.00			
10. Age	–.05	.09	–.02	–.18**	–.05	.07	–.06	–.06	–.06	1.00		
11. Education (high)	.08	.03	.24***	.12*	–.21***	–.04	–.01	–.01	.02	–.05	1.00	
12. Sector (private)	.31***	–.09	.12*	–.01	–.05	.04	–.14*	.00	.11†	.23***	.28***	1.00
Mean	4.15	3.71	3.54	3.28	3.83	2.64	3.85	5.79	1.71	48.93	.80	.78
Standard deviation	.39	.56	.43	.59	.45	.78	.96	.89	.45	10.56	.40	.41

*** $p<.001$, ** $p<.01$, * $p<.05$, † p<.10.
