# Supplementary material for: Does Personality Have a Different Impact on Self-Rated Distraction, Job Satisfaction, and Job Performance in Different Office Types?
Source: PLoS One. 2016 May 25;11(5):e0155295. doi: 10.1371/journal.pone.0155295 (PMC4880328; doi:10.1371/journal.pone.0155295)
Supplement: S2 Table — (PDF) [file pone.0155295.s002.pdf]

S2 Table. Correlations between the personality traits and outcome variables (*n*=89), shared office type.

	1.	2.	3.	4.	5.	6.	7.	8.	9.	10.	11.	12.
1. Agreeableness	1.00											
2. Emotional stability	.30**	1.00										
3. Openness to experience	.33**	.21*	1.00									
4. Extraversion	.38***	.33**	.34**	1.00								
5. Conscientiousness	.41***	.11	.11	.15	1.00							
6. Distraction	–.08	–.33**	.02	–.14	.06	1.00						
7. Job satisfaction	.18†	.31**	–.03	.17	.20†	–.24*	1.00					
8. Professional efficacy	.15	.29**	.32**	.21*	.27*	–.00	.54***	1.00				
9. Gender (female)	.19†	–.20†	–.01	–.12	.20†	.11	.15	.07	1.00			
10. Age	.01	.22*	–.01	–.10	–.07	.01	.03	.10	.10	1.00		
11. Education (high)	.15	–.09	.23*	–.08	.04	.13	–.02	.05	.16	–.01	1.00	
12. Sector (private)	.14	.11	.05	–.06	–.02	.01	.02	.05	.20†	.31**	.27*	1.00
Mean	4.21	3.74	3.62	3.30	3.78	3.32	3.78	5.92	1.73	45.92	.88	.92
Standard deviation	.42	.61	.45	.58	.48	.91	1.06	.83	.45	9.87	.33	.27

*** *p*<.001, ** *p*<.01, * *p*<.05, † p<.10.
