# Supplementary material for: Does Personality Have a Different Impact on Self-Rated Distraction, Job Satisfaction, and Job Performance in Different Office Types?
Source: PLoS One. 2016 May 25;11(5):e0155295. doi: 10.1371/journal.pone.0155295 (PMC4880328; doi:10.1371/journal.pone.0155295)
Supplement: S3 Table — (PDF) [file pone.0155295.s003.pdf]

S3 Table. Correlations between the personality traits and outcome variables (*n*=784), open office type.

	1.	2.	3.	4.	5.	6.	7.	8.	9.	10.	11.	12.
1. Agreeableness	1.00											
2. Emotional stability	.18***	1.00										
3. Openness to experience	.23***	.05	1.00									
4. Extraversion	.22***	.16***	.36***	1.00								
5. Conscientiousness	.27***	.20***	–.00	.00	1.00							
6. Distraction	.15***	–.28***	.15***	.00	.01	1.00						
7. Job satisfaction	.16***	.29***	.03	.08*	.13***	–.24***	1.00					
8. Professional efficacy	.26***	.30***	.11**	.12***	.28***	–.03	.35***	1.00				
9. Gender (female)	.30***	–.10**	–.08*	.04	.14***	.13***	.03	.04	1.00			
10. Age	–.03	.18***	–.09*	–.12***	.04	–.02	.04	.07†	–.09**	1.00		
11. Education (high)	.01	–.10**	.20***	.14***	–.10**	.10**	–.07†	–.05	–.02	–.21***	1.00	
12. Sector (private)	.18***	–.03	.07*	.02	.01	.15***	–.01	–.04	.21***	.07*	.10**	1.00
Mean	4.06	3.66	3.51	3.26	3.77	3.48	3.85	5.86	1.58	47.11	.70	.68
Standard deviation	.46	.60	.51	.59	.46	.86	.93	.85	.49	10.77	.46	.47

*** $p<.001$, ** $p<.01$, * $p<.05$, † $p<.10$.
