# Supplementary material for: Does Personality Have a Different Impact on Self-Rated Distraction, Job Satisfaction, and Job Performance in Different Office Types?
Source: PLoS One. 2016 May 25;11(5):e0155295. doi: 10.1371/journal.pone.0155295 (PMC4880328; doi:10.1371/journal.pone.0155295)
Supplement: S4 Table — (PDF) [file pone.0155295.s004.pdf]

S4 Table. Correlations between the personality traits and outcome variables (*n*=57), flex office type.

	1.	2.	3.	4.	5.	6.	7.	8.	9.	10.	11.	12.
1. Agreeableness	1.00											
2. Emotional stability	–.09	1.00										
3. Openness to experience	.07	.03	1.00									
4. Extraversion	.33*	.14	.04	1.00								
5. Conscientiousness	.09	.06	.19	.19	1.00							
6. Distraction	.25†	–.48***	–.10	–.06	.15	1.00						
7. Job satisfaction	–.22†	.44***	.16	.01	–.05	–.54***	1.00					
8. Professional efficacy	.02	.37**	.35**	.10	.20	–.25†	.34**	1.00				
9. Gender (female)	.11	–.29*	–.20	–.09	–.26†	.13	.02	–.22†	1.00			
10. Age	–.22	–.01	–.04	–.14	.05	–.05	–.02	.16	.12	1.00		
11. Education (high)	.14	–.10	–.02	–.01	–.15	–.07	–.01	–.14	.24†	–.02	1.00	
12. Sector (private)	.37**	.02	.06	.10	–.20	.03	.20	–.03	.25†	.12	.33*	1.00
Mean	4.06	3.67	3.54	3.31	3.69	3.29	3.84	5.83	1.46	47.05	.82	.33
Standard deviation	.39	.61	.46	.46	.40	.89	.88	.83	.50	9.95	.38	.48

*** $p<.001$, ** $p<.01$, * $p<.05$, † p<.10.
